# Selenium Nanoparticle Synthesized by *Proteus mirabilis* YC801: An Efficacious Pathway for Selenite Biotransformation and Detoxification

**DOI:** 10.3390/ijms19123809

**Published:** 2018-11-29

**Authors:** Yuting Wang, Xian Shu, Jinyan Hou, Weili Lu, Weiwei Zhao, Shengwei Huang, Lifang Wu

**Affiliations:** 1 Key Laboratory of High Magnetic Field and Ion Beam Physical Biology, Hefei Institute of Physical Science, Chinese Academy of Sciences, Hefei 230031, China; wangyuting2006@outlook.com (Y.W.); sx360775419@gmail.com (X.S.); jyhou@ipp.ac.cn (J.H.); luweili@ahmu.edu.cn (W.L.); annyzhao@ipp.ac.cn (W.Z.); 2 The Sericultural Research Institute, Anhui Academy of Agricultural Science, Hefei 230061, China; 3 School of Life Sciences, University of Science and Technology of China, Hefei 230026, China; 4 Key Laboratory of Environmental Toxicology and Pollution Control Technology of Anhui Province, Hefei Institutes of Physical Science, Chinese Academy of Sciences, Hefei 230031, China; 5 Anhui Key Laboratory of Bioactivity of Natural Products, School of Pharmacy, Anhui Medical University, Hefei 230032, China

**Keywords:** biogenic selenium nanoparticles, selenite reduction, *Proteus mirabilis* YC801, electron microscopy analysis, Fourier Transform Infrared (FTIR) Spectroscopy, Real-time PCR

## Abstract

Selenite is extremely biotoxic, and as a result of this, exploitation of microorganisms able to reduce selenite to non-toxic elemental selenium (Se^0^) has attracted great interest. In this study, a bacterial strain exhibiting extreme tolerance to selenite (up to 100 mM) was isolated from the gut of adult *Monochamus alternatus* and identified as *Proteus mirabilis* YC801. This strain demonstrated efficient transformation of selenite into red selenium nanoparticles (SeNPs) by reducing nearly 100% of 1.0 and 5.0 mM selenite within 42 and 48 h, respectively. Electron microscopy and energy dispersive X-ray analysis demonstrated that the SeNPs were spherical and primarily localized extracellularly, with an average hydrodynamic diameter of 178.3 ± 11.5 nm. In vitro selenite reduction activity assays and real-time PCR indicated that thioredoxin reductase and similar proteins present in the cytoplasm were likely to be involved in selenite reduction, and that NADPH or NADH served as electron donors. Finally, Fourier-transform infrared spectral analysis confirmed the presence of protein and lipid residues on the surfaces of SeNPs. This is the first report on the capability of *P. mirabilis* to reduce selenite to SeNPs. *P. mirabilis* YC801 might provide an eco-friendly approach to bioremediate selenium-contaminated soil/water, as well as a bacterial catalyst for the biogenesis of SeNPs.

## 1. Introduction

Selenium (Se) is of considerable environmental importance, as it is an essential micronutrient with a prominent role in the health of various species [1,2]. For example, Se is required as a cofactor for numerous proteins, such as glutathione peroxidases and thioredoxin reductases in animals, and thus plays a pivotal role in removing the products of damage caused by free radicals and reactive oxygen species [3,4]. Moreover, selenium supplementation can significantly increase the activities of both selenium-dependent glutathione peroxidase (Se-GSH-Px) and superoxide dismutase [5,6]. Overexposure to Se can disrupt protein integrity and reduce cellular enzymatic activity, leading to apoptotic cell death; this results in chronic or acute selenosis [7,8]. Therefore, Se is of significant research interest for both environmental and public health purposes due to its complicated characteristics; the difference between essential and toxic levels of selenium is simply one order of magnitude [9]. Selenium occurs in the environment in a variety of redox states, including VI (selenate), IV (selenite), 0 (elemental selenium), -II (selenide), and organic selenium species (e.g., methylated compounds and seleno amino acids). Among them, selenite (SeO_3_^2−^) and selenate (SeO_4_^2−^) show the greatest biotoxic effects due to their high solubilities and bioavailabilities. Elemental selenium (Se^0^) is the least mobile form and cannot readily be used by biological systems; Se^0^ is generally considered biologically inert and safe for terrestrial and aquatic environments in low amounts [10]. Therefore, biogeochemical cycles that involve the reduction of selenite/selenate to Se^0^ are of paramount importance and have attracted worldwide attention [9,11].

Although several methods, including chemical precipitation, catalytic reduction, and adsorption/ion exchange, have been proposed to reduce the concentrations of Se oxyanions in natural and environmental waters [12,13,14], limitations such as the generation of large volumes of sludge or high chemical reagent costs have made them less desirable [1]. However, biological approaches are generally preferred due to additional benefits, such as their eco-friendly characteristics, as well as their abilities to employ self-generating catalysts. They can provide a viable and cost-effective approach for bioremediation of excess selenium in contaminated water [14]. Thus, the role of microorganisms in the geological cycle of this element is important. Microbial conversion of SeO_3_^2−^ to Se^0^ is widely recognized as a detoxification strategy, whereby the toxic and soluble oxyanion is converted to solid Se^0^. The reduction of SeO_4_^2−^/SeO_3_^2−^ to Se^0^ has been observed in a range of microorganisms under aerobic and anaerobic growth conditions, such as *Enterobacter cloacae* [15], *Pseudomonas putida* [16], *Duganella* sp. [8], *Agrobacterium* sp. [8], *Azoarcus* sp. CIB [17], *Bacillus mycoides* [18], and *Saccharomyces cerevisiae* [19]. For example, Watts et al. [20] reported that selenate reduction by *E. cloacae* SLD1a-1 was catalyzed by a molybdenum-dependent membrane-bound enzyme and that the enzyme activity can be enhanced by 1 mM sodium molybdate; however, it is significantly reduced by 1 mM sodium tungstate. Lampis et al. [18] found that *B. mycoides* was able to reduce 0.5 and 2.0 mM SeO_3_^2−^ within 12 and 24 h respectively; moreover, spherical-shaped Se nanoparticles (SeNPs) were mostly observed outside the bacterial cell and were rarely present in the cytoplasmic compartment. Research by Kieliszek et al. [2] regarding the accumulation and metabolism of selenium in *S. cerevisiae* MYA-2200 and *Candida utilis* ATCC 9950 demonstrated that selenium supplementation can increase the proportion of unsaturated acids (e.g., linoleic acid and linolenic acid) in the biomass of both yeast strains, and that it warrants further attention as a potential source of protein-selenium preparations. Finally, the formation of selenium nanoparticles in yeast cells grown in the presence of selenium was also observed by single-particle inductively coupled plasma mass spectrometry (ICPMS) [21].

Interestingly, some microorganisms link their selenite reduction capability to the biosynthesis of SeNPs, which can accumulate intracellularly or be deposited extracellularly. Due to their unique physical and chemical properties, SeNPs have some unusual advantages in biosensors, bioremediation, biomedical therapy, and environmental remediation [22,23,24,25,26]. Furthermore, SeNPs were found to strongly inhibit the growth of the key human pathogenic bacterium, *Staphylococcus aureus* [27]. Compared with classical chemical synthesis methods, synthesis of SeNPs by microorganisms is unique and more ecologically friendly, primarily because those microorganisms constitute inexpensive catalysts, avoid the use of hazardous reducing agents, and facilitate reproducibility in production, as well as easy scaling-up [28,29]. Therefore, microorganisms are attractive as theoretical nanofactories for the green production of nano-selenium. However, the tolerance of most known bacterial strains to SeO_4_^2−^/SeO_3_^2−^ is relatively low, and the time necessary for the degradation of these toxic forms is relatively long; thus, new strains with high Se tolerance or rapid Se metabolic ability are urgently needed.

Here, a gut bacterium, *Proteus mirabilis* YC801, was isolated from the gut of the adult form of the herbivorous insect *Monochamus alternatus* (Coleoptera: Cerambycidae) and revealed to exhibit a high tolerance to SeO_3_^2−^. *P*. *mirabilis* strains are widely distributed in soil and water in the natural environment, as well as in animal guts [30]. Moreover, the *P. mirabilis* strains possess a vast range of metabolic activities toward heavy metals or toxic substances, allowing their adaptation to different environmental conditions [31,32], and thus providing a possibility of employing these microorganisms in bioremediation and environmental protection. Therefore, the use of *P*. *mirabilis* strain YC801 as a bacterial chassis may provide a new, rapid, non-hazardous, and easily scaled-up biosynthetic pathway for selenite biotransformation and SeNPs production. In this study, we investigated selenite reduction and its possible molecular mechanism, as well as SeNP biosynthesis, by *P*. *mirabilis* strain YC801.

## 2. Results and Discussion

### 2.1. Isolation, Characterization, and Identification of Bacterial Strains

**Figure 1:**
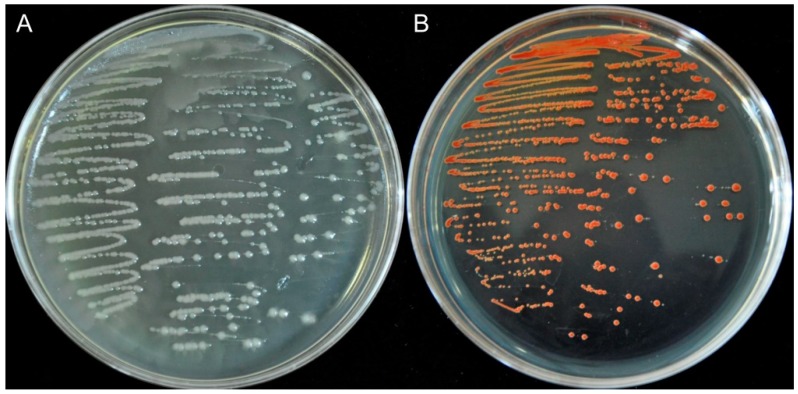
Growth of strain YC801 on YEP (yeast extract peptone) agar plates without (**A**) and with (**B**) 5.0 mM selenite. The red colony color indicates that the strain reduced selenite to elemental red selenium (Se^0^).

Insect species are known to harbor rich and complex microbial communities in their intestinal tracts, which may participate in many relationships with their hosts [33,34]. In the present study, 28 bacterial strains were isolated from the gut of the adult form of *M. alternatus*, using yeast extract peptone (YEP) plates which contained 10 mM sodium selenite. These results further confirm that the insect gut could be a good source of novel microorganisms which are able to effectively reduce selenite to elemental selenium (Se^0^). Of the 28 strains, isolate #801 grew well on selenite media and exhibited the capability to reduce selenite to red Se^0^ (Figure 1). Therefore, this isolate (YC801) was chosen for further study.

**Figure 2:**
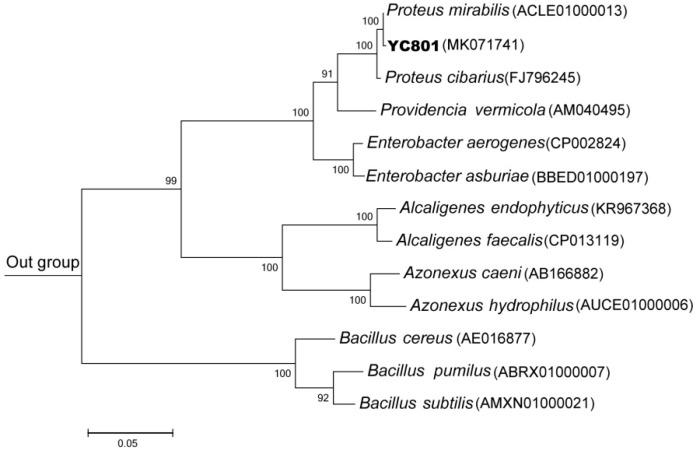
A maximum likelihood tree based on the *16S rRNA* gene sequence of isolate YC801 and related representative strains. The scale bars indicate 0.05 substitutions per site. *Sphingobacterium zeae* (KU201960) was used as the outgroup.

A 1496-bp fragment of the *16S rRNA* gene of the strain was obtained, and its sequence was most similar to that of *P. mirabilis* ATCC 29906(T) (99.86%). Phylogenetic analysis based on the maximum likelihood method also placed isolate YC801 within the same cluster as *P. mirabilis* ATCC 29906(T) (Figure 2). More importantly, the results of physiological and biochemical tests showed that the isolate YC801 possessed biochemical and physiological characteristics typical of *P. mirabilis* (Table 1). Thus, strain YC801 was identified as *P. mirabilis* YC801. Previous studies have shown that *P. mirabilis* possesses high resistance to heavy metals, antibiotics, and aromatic hydrocarbons [31]. However, this is the first study to demonstrate that a *P. mirabilis* strain can biosynthesize SeNPs by the reduction of selenite to Se^0^.

### 2.2. Bioconversion of SeO_3_^2−^ And Elemental Selenium Formation

To study the potential of *P*. *mirabilis* YC801 to be used in bioremediation of contaminated wastewaters and soils, we performed minimum inhibitory concentration (MIC) assays to determine its selenite tolerance. The isolate YC801 showed high tolerance towards selenite with a MIC of 100 mM, much higher than those described in previous reports regarding *Bacillus thermoamylovorans* (8 mM) [35], *Rhizobium* sp. (16 mM) [36], and *Streptomyces* sp. (50 mM) [37]; however, it was similar to that of other highly selenite-resistant bacteria, including *Rahnella aquatilis* (85 mM) [38] and *Vibrio natriegens* (100 mM) [11].

**Figure 3:**
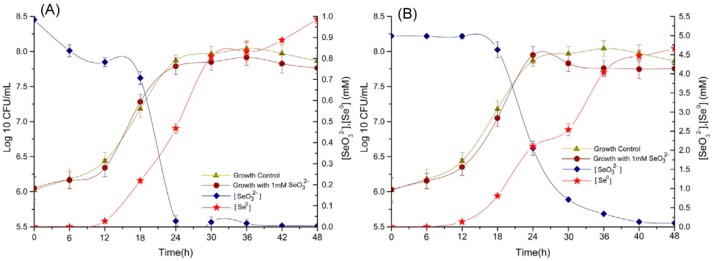
Bacterial growth curve, SeO_3_^2−^ reduction, and Se^0^ production by the strain *P. mirabilis* YC801 cultured in YEP medium containing (**A**) 1 mM; and (**B**) 5.0 mM of Na_2_SeO_3_. Each test was performed in triplicate and data were presented with standard deviations.

The ability of *P. mirabilis* YC801 to reduce selenite was studied in liquid yeast extract peptone (YEP) medium supplemented with 1.0 and 5.0 mM Na_2_SeO_3_, respectively. The results are shown in Figure 3. Overall, both concentrations of selenite did not cause severe toxicity in this bacterial strain, because the relative growth curves followed the same pattern as that of the control culture, which did not contain selenite. Moreover, SeO_3_^2−^ reduction and Se^0^ formation by *P. mirabilis* YC801 appeared to be a single continuous process that was associated with the growth kinetics of the strain. In *P. mirabilis* YC801 cultures with an initial selenite concentration of 1.0 mM, most of the remaining selenite (>81%) was exhausted during the exponential growth phase (between 12 and 24 h); it was completely reduced after 42 h of incubation (Figure 3A), although 16.3% of the SeO_3_^2−^ had been reduced within 6 h. Notably, reduction required additional time in the presence of 5 mM selenite; depletion of SeO_3_^2−^ was scarcely observed within 12 h at the SeO_3_^2−^ concentration of 5.0 mM (only 0.4% of the SeO_3_^2−^ was reduced at this stage). However, selenite reduction occurred at the very end of the exponential growth phase and well into the stationary phase. During the exponential phase, only 59% of the initial selenite was reduced, whereas the remaining selenite was depleted during the stationary phase after 48 h of incubation (Figure 3B). Lower amounts of selenite were reduced more quickly by *P. mirabilis* YC801 than were higher selenite concentrations; this phenomenon was also reported in *Burkholderia fungorum* [39], *B. mycoides* [18], and *Stenotrophomonas maltophilia* [40]. For example, Lampis et al. [40] found that SeO_3_^2−^ reduction and Se^0^ formation by *S. maltophilia* SeITE02 were associated with the growth kinetics of the strain. When *S. maltophilia* SeITE02 grew in culture medium supplemented with a low concentration of selenite (0.5 mM), 100% of the selenite was consumed after 52 h of incubation (this primarily occurred during the exponential phase). However, when grown in culture medium with 2.0 mM selenite, less than 10% of the SeO_3_^2−^ was reduced after 52 h of incubation, whereas 86% of the total selenite was reduced after 192 h of incubation (this primarily occurred during the stationary phase). Wang et al. [41] also reported that *Alcaligenes faecalis* Se03 showed different behaviors in the presence of 1 mM or 5 mM selenite. In cultures with an initial selenite concentration of 1 mM, approximately >90% of SeO_3_^2−^ exhaustion occurred during the exponential growth phase (between 12 and 24 h); conversely, in the presence of 5 mM selenite, the greatest reduction (>73%) occurred at the very end of the exponential growth phase and stretched into the stationary phase (between 18 and 42 h). Therefore, selenite reduction behavior by *P. mirabilis* YC801 is most likely related to the initial selenite amount of the culture and/or the bacterial growth phase; this observation supports the assumption that selenite reduction of this strain is accomplished by cellular reductases and/or reducing compounds, whose production and consumption capacities are linked to the growth phase of the microbe [28].

Numerous microorganisms are known to be capable of a range of transformations of selenium compounds, including reduction, methylation, oxidation, and demethylation [22]. For example, two yeast strains, *S. cerevisiae* MYA-2200 and *C. utilis* ATCC 9950, can bioaccumulate selenite in various cell structures, and then convert it into organic forms (such as selenomethionine, selenomethionine-NH_3_, and 2,3-DHP-selenocysteine-cysteine); this capability was detected by high-performance liquid chromatography–inductively coupled plasma mass spectrometry (HPLC-ICP-MS) and ultra-high-performance liquid chromatography-electrospray ionization (UHPLC-ESI)–orbitrap mass spectrometry techniques [42,43]. The metal-reducing bacterium *Veillonella atypica* was capable of transforming selenite to selenide in anaerobic environments [44]. However, some bacteria, such as *E. cloacae* [15], and *P. putida* [16] are able to transform selenite to Se^0^. Kieliszek et al. [19] also reported that yeast cells could reduce selenite to elemental selenium in the presence of sulfate (IV) ions. In the present study, the reduction of SeO_3_^2−^ was accompanied by the appearance of a bright red color in growth medium supplemented with Na_2_SeO_3_, indicating the production of Se^0^. Approximately 95% of the selenite in the culture medium was reduced and subsequently converted to Se^0^ at both concentrations tested. Significantly more time was required to completely deplete SeO_3_^2−^ in the presence of 5.0 mM SeO_3_^2−^ (Figure 3B). However, no visible color change was observed in flasks containing only selenite or only the bacterial strain, indicating that this bacterial strain actively participated in selenite reduction and Se^0^ formation. 

Although the selenite reduction process runs parallel to that of microbial growth, red color in cell suspensions appeared later; the bacterial cultures turned light red after 6 and 18 h of incubation at selenite concentrations of 1.0 and 5.0 mM, respectively (Figure 3). The delayed formation of red elemental selenium was in good agreement with the Se^0^ levels in bacterial cultures, as determined by spectrophotometry in this study. In particular, at a concentration of 1.0 mM SeO_3_^2−^, 16.3% of the initial selenite was reduced after 6 h of incubation, whereas only 5.8% was transformed into detectable Se^0^. The delay in the formation of Se^0^ has been reported in similar studies involving other bacteria, such as *B. mycoides* [18], *S. maltophilia* [40], and *A. faecalis* [41]. Lampis et al. [18] reported delays of 6 and 9 h between the depletion of SeO_3_^2−^ and the detection of elemental selenium when *B. mycoides* was cultured in growth medium supplemented with 0.5 mM and 2.0 mM SeO_3_^2−^, respectively. Delayed formation of elemental selenium, with regard to selenite reduction, was also found in *B. fungorum* DBT1 cultured in nutrient broth containing 2 mM selenite [39]. All of these results indicated that SeO_3_^2−^ is likely converted to an intermediate reduced-Se form, such as organic selenide (RSeR), prior to its ultimate reduction to Se^0^, as suggested by Van Fleet-Stalder et al [45]. 

### 2.3. Localization and Characterization of Selenium Nanoparticles

**Figure 4:**
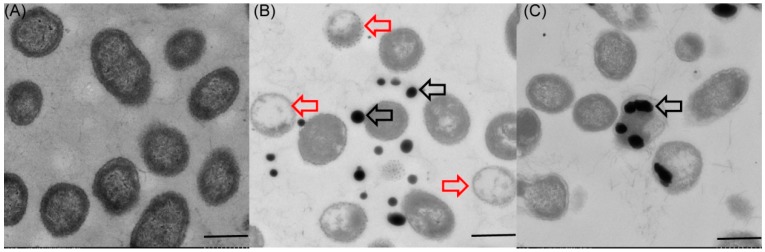
Transmission electron microscopy (TEM) images of *P. mirabilis* YC801 grown without Na_2_SeO_3_ (**A**) and with 5 mM Na_2_SeO (**B**,**C**) after 36 h incubation. Electron-dense nanoparticles (white arrows) are located extracellularly (**B**) or intracellularly (**C**). The red arrows indicate “empty ghost” cells. The bar represents 1 μm.

**Figure 5:**
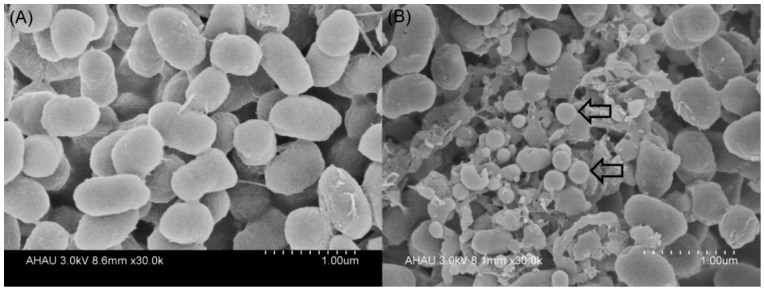
SEM micrographs of *P. mirabilis* YC801 grown without Na_2_SeO_3_ (**A**) and with 5 mM Na_2_SeO (**B**) after 36 h incubation. Electron-dense nanoparticles are located extracellularly as indicated by white arrows.

Because a number of bacteria have the ability to mediate reduction of sodium selenite into SeNPs, we investigated whether *P. mirabilis* YC801 can reduce selenite to Se^0^ during formation of SeNPs. Transmission electron microscopy (TEM) revealed that the majority of the SeNPs were distributed outside the cell, and few were found in the cytoplasm (Figure 4B,C). In the control group (grown without sodium selenite), no such particles were detected (Figure 4A). The particles were spherical and of various sizes (i.e., diameters ranging from approximately 100–400 nm), similar to those produced by *B. mycoides* (50–400 nm) [18], *P. putida* (100–500 nm) [16], and *V. natriegens* (100–400 nm) [11]. Furthermore, selenium nanoparticles appeared extracellularly to a noticeable extent, with different sizes detected by scanning electron microscopy (SEM) (Figure 5, indicated by white arrows). Notably, TEM analysis also detected empty ghost cells (indicated by red arrows in Figure 4B). An indicator of cell membrane breakage and cytoplasm leakage, empty ghost cells were also found in a study of *C. utilis* ATCC 9950, when the yeast was cultured in medium supplemented with sodium selenite, particularly in the stationary phase [46]. A study regarding the bacterial biogenesis of SeNPs by *S. maltophilia* also revealed that empty cells were abundant during the stationary growth phase of the bacterium in the presence of 0.5 mM selenite, possibly due to the release of Se^0^ particles into the medium [40]. Therefore, cell lysis may be involved in the release of intracellularly generated SeNPs by *P. mirabilis* YC801. However, more research is needed to elucidate mechanisms involved in the release of Se^0^ nanoparticles.

### 2.4. Characterization of SeNPs Produced by *P. mirabilis* YC801

#### 2.4.1. Dynamic Light Scattering (DLS) Analyses and SEM- (Energy Dispersive X-ray) Analysis (EDX)

**Figure 6:**
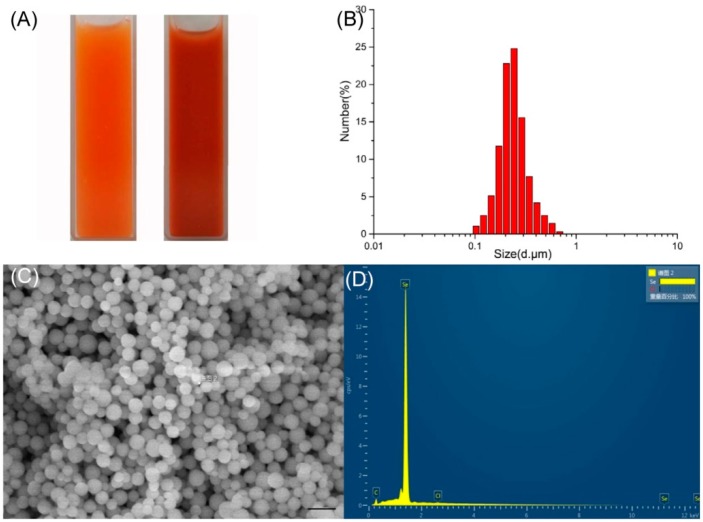
Dynamic Light Scattering (DLS) spectra and SEM-EDX (Energy Dispersive X-ray) analysis of purified SeNPs produced by *P. mirabilis* YC801 grown with 5.0 mM selenite. (**A**) Selenite-dosing cells (left) and purified nano-selenium (right); (**B**) DLS spectra of purified SeNPs; (**C**) SEM analysis for purified SeNPs; (**D**) EDX spectra for purified SeNPs. The bar represents 1 μm.

SeNPs from cultures of *P. mirabilis* YC801 were spherical, and DLS analysis revealed an average dimension of 178.3 ± 11.5 nm (Figure 6). Similar size distributions of SeNPs have been found in other bacteria, such as *Rhodopseudomonas palustris* (165 nm) and *V. natriegens* (136 ± 31 nm) [11]. Fernández-Llamosas et al. [11] also found that the *Azoarcus* sp. CIB could reduce selenite, both aerobically and anaerobically. However, in the present study, SeNPs obtained using the anaerobic culture method were smaller than those obtained using the aerobic culture method (90 ± 26 nm and 174 ± 36 nm, respectively). In addition, subsequent EDX analysis of the nanoparticles showed only Se with traces of carbon and Cl. The electron-dense particles produced specific selenium absorption peaks at 1.37 keV (peak SeLa), 11.22 keV (peak SeKa), and 12.49 keV (peak SeKb).

#### 2.4.2. Fourier-Transform Infrared Spectroscopy (FTIR) Analysis

**Figure 7:**
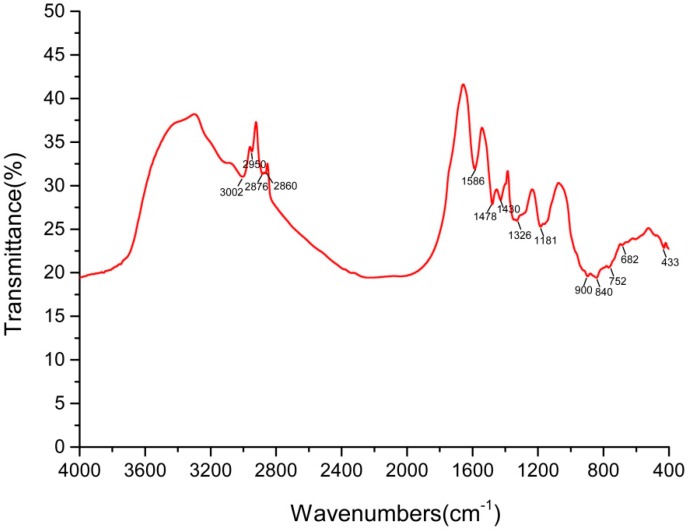
The FTIR spectrum of SeNPs synthesized by isolate YC801.

The FTIR spectrum of the SeNPs produced by *P. mirabilis* YC801 is shown in Figure 7. The various absorption bands in the FTIR spectra and their corresponding assignments are included below; the band recorded near 3000 cm^−1^ was due to C-H broad alkyl stretching. Asymmetric stretching of C-H bonds in the methyl groups of both aliphatic and aromatic compounds appeared at 2940 cm^−1^, while peaks at 2876 cm^−1^ were assigned to asymmetric and symmetric vibrations of the methyl groups. The peaks at 2860 cm^−1^ corresponded to the symmetric and antisymmetric stretching vibrations of -CH_2_. Peaks at 1586 cm^−1^ were assigned to the C=C stretching of the quinoid and benzenoid rings. The spectra showed also symmetric NH_3_^+^-deformation vibrations and symmetric COO-stretching vibrations at 1478 cm^−1^ and 1430 cm^−1^, respectively. The line at 1326 cm^−1^ was assigned to lipid content, C-H band vibrations, or syringyl ring breathing with C=O stretching. Furthermore, peaks at 1181, 900, and 840 cm^−1^ were mainly attributed to C=O stretching vibrations, anomeric carbon vibration, and C-H in-plane benching, respectively. Finally, the presence of a peak at 752 cm^−1^ was attributed to the ring-vibrating modes of ortho-substituted aromatics. Thus, the results clearly indicated the presence of organic residues, such as carbohydrates, lipids, and proteins, on the surface of SeNPs produced by *P. mirabilis* YC801. Lampis et al. [40] found that various organic residues were present on the surface of the biogenic SeNPs produced by *S. maltophilia*, but were completely absent in SeNPs synthesized chemically. These biochemicals were suspected to be involved in the formation or stabilization process of biogenic SeNPs. Debieux et al. [47] also reported that Se nanospheres produced by *Thauera selenatis* were stabilized under the presence of a protein of approximately 95 kDa, Se factor A (SefA). Dobias et al. [48] identified four proteins (AdhP, Idh, OmpC, and AceA) that bound specifically to biogenic SeNPs, all of which played a critical role in controlling particle size and morphology of SeNPs. Therefore, our findings indicate that bacterial proteins may be involved in selenite reduction; additionally, they corroborate the synthesis and stabilization data reported regarding SeNPs.

### 2.5. Biocatalytic Selenite Reduction Activity Assays

**Figure 8:**
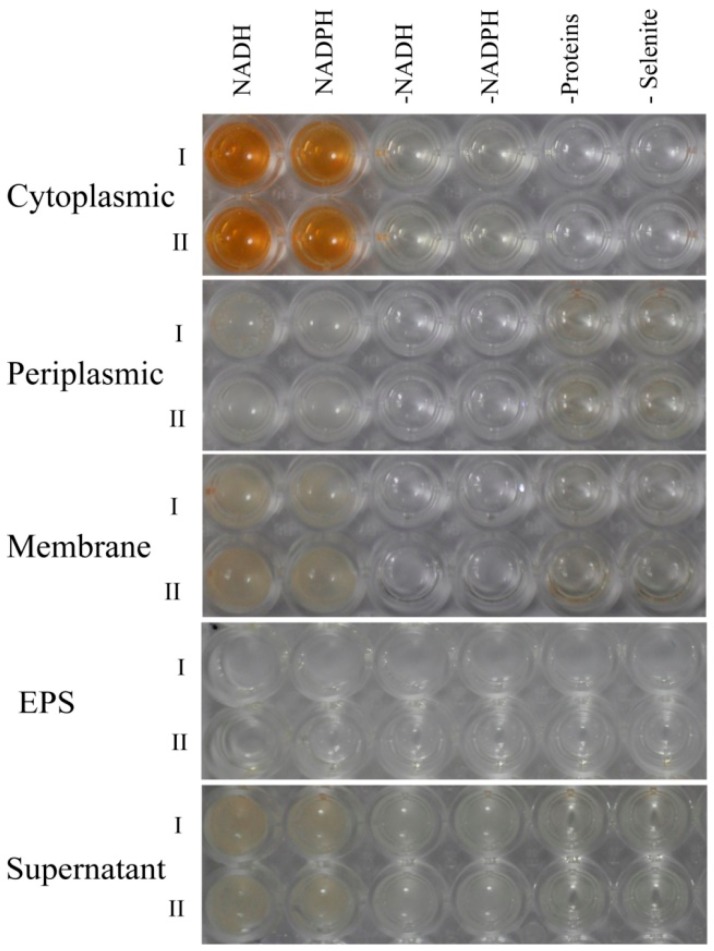
Enzymatic assay on different cell compartments (cytoplasmic, periplasmic, and membrane fractions) or culture broth fractions (supernatant, and exopolysaccharide) with selenite reduction activity. All assays were done in duplicate (indicated by roman numerals), with the addition of 5.0 mM SeO_3_^2−^ and 2.0 mM NADH or NADPH. Three negative controls were also used: Without protein fractions or culture broth fractions, without selenite, and without NADH or NADPH.

Extracellular selenite and selenite are suspected to be transported across the plasma membrane by high-affinity sulfate transporters [48], or a nonspecific transport system (such as nitrate transporters and the DedA protein) [49,50]. The reduction of SeO_3_^2−^ to Se^0^ in microorganisms is then achieved by various mechanisms, such as Painter-type reactions [51], thiol-containing or glutathione reductases, fumarate reductase [16,52], and sulfide- and siderophore-mediated reduction [53]. Because various enzymatic systems are reportedly involved in the reduction of selenite and production of SeNPs in bacteria, an in vitro enzymatic selenite reduction assay was conducted to clarify the mechanism of selenite reduction to biogenic SeNPs in *P. mirabilis* YC801. The selenite reduction activity of different subcellular fractions of the cell culture is presented in Figure 8; the results clearly indicated that selenite reduction activity in *P. mirabilis* YC801 cells is localized in the cytoplasmic fraction. However, this reduction solely occurs in the presence of an electron donor (NADH or NADPH), suggesting that reduction in the cytoplasmic fraction is an enzymatic process. Previous studies of selenite reduction in two *B. fungorum* strains (*B. fungorum* DBT1 and *B. fungorum* 95) have shown that selenite reduction occurred mainly in the cytoplasmic fractions only after addition of an electron donor (NADH or NADPH). Furthermore, significant selenite reduction activity was detected in the cytoplasmic fraction of *S. maltophilia* SeITE02 cells, with NADH serving as an electron donor, while no reduction activity was found in the exopolysaccharide (EPS) fraction or membrane fraction [40]. Thus, selenite reduction by *P. mirabilis* YC801 is likely to occur through cytoplasmic enzymatic reduction with the involvement of reductases, such as thioredoxin reductase [54], glutathione reductase [52], or NADH/NADPH-related reductases [55]. However, TEM analysis revealed that selenium nanoparticles were primarily present in the extracellular space after 36 h incubation (Figure 4B), suggesting that the SeNPs are generated inside the cell and then released into the extracellular space through a currently unknown export system, such as outer membrane vesiculation or cell lysis [41]. Therefore, additional experiments should be performed to identify both the nature of Se^0^ formation and the release mechanism of SeNPs produced by *P. mirabilis* YC801.

### 2.6. Real-Time PCR(qPCR) Analysis

**Figure 9:**
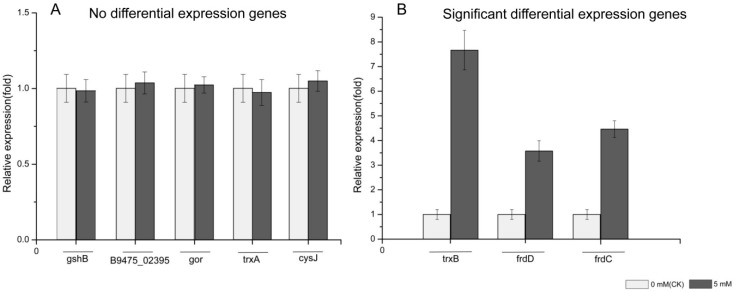
Transcript levels of selected genes quantified by Real-time PCR. (**A**) Non-differentially expressed genes; (**B**) significant differentially expressed genes. Data are shown as fold changes by calculating transcript levels of selenite treated samples compared to untreated (defined as 1). Data represent an average of three biological replicates ± SD.

Because in vitro enzymatic selenite reduction analysis showed that selenite reduction in the cytoplasmic fraction of *P. mirabilis* YC801 cells is an enzymatic reaction, rather than a chemically catalyzed reaction, mRNA expression levels of genes suspected to be involved in selenite reduction and SeNP formation in *P. mirabilis* YC801 were assessed using qPCR. Eight candidate genes, including glutathione synthetase (*gsh*B), thioredoxin reductase (*trx*B), nitrite reductase (NAD(P)H) (*B9475_02395*), glutathione reductase (*gor*), fumarate reductase subunit D (*frd*D), fumarate reductase subunit C (*frd*C), thioredoxin (*trx*A), and sulfite reductase [NADPH] flavoprotein alpha-component (*cys*J) were chosen (Table 2). The mRNA expression levels of these genes are shown in Figure 9. As determined by qPCR, the mRNA expression levels of *gsh*B, *B9475_02395*, *gor*, *trx*A, and *cys*J in cells cultured with selenite were not different from those in cells without selenite treatment, suggesting that GSH, nitrite reductase, and sulfite reductase might not be involved in the reduction of selenite to SeNPs by *P. mirabilis* YC801. However, selenite treatment significantly increased the mRNA expression levels of *trx*B, *frd*D, and *frd*C (by 7.6-fold, 3.6-fold, and 4.4-fold, respectively). Sodium selenite is a substrate for the thioredoxin system and can be reduced to Se^0^ by thioredoxin reductase in *Escherichia coli* [56], *Bacillus subtilis* [57], and *Pseudomonas seleniipraecipitans* [58]. Moreover, Song et al. [15] reported that *E. cloacae* Z0206 can completely reduce selenite to Se^0^ and form SeNPs under aerobic conditions through a fumarate reductase-mediated mechanism, while Kieliszek et al. [19] suggested that fumarate reductase performs selenite reduction by receiving electrons from NADH dehydrogenase and the quinol pool through cytochrome C [59].

Overall, these data suggest that selenite may be reduced to Se^0^ via the actions of reductases, such as thioredoxin reductase and fumarate reductase, instead of GSH-mediated Painter-type reactions. However, it is likely that multiple enzymes (mechanisms) are involved in the reduction of selenite and biogenesis of SeNPs by *P. mirabilis* YC801, and there remains debate regarding the possibility of parallel function or synergistic action. Therefore, additional research, such as proteomic analysis or mutant construction, is needed to further clarify possible reduction mechanisms in *P. mirabilis* YC801.

## 3. Materials and Methods

### 3.1. Chemicals and Culture Medium

YEP broth was purchased from Shanghai Rui Chu Bio-Tech Co., Ltd. (Shanghai, China). Sodium selenite (Na_2_SeO_3_, ≥99%) was purchased from Sigma-Aldrich (St. Louis, MO, USA). For the preparation of Na_2_SeO_3_ stock solution (2 mol/L), Na_2_SeO_3_ was diluted into distilled deionized water (ddH_2_O) and filter-sterilized. All other analytical grade reagents were purchased from Sangon Biotech Co., Ltd. (Shanghai, China) and Sinopharm Chemical Reagent Co., Ltd. (Shanghai, China). 

### 3.2. Bacterial Strain Isolation and Identification

Recently emerged *M. alternatus* (Coleoptera: Cerambycidae) adults were collected from *Pinus massoniana* that grow in Chizhou, Anhui Province, China. The insects were then dissected under a dissecting microscope. The extracted guts were pooled and homogenized, as described by Huang et al. [60] with minor modification. Briefly, 1 g of homogenized gut sample was diluted serially (10-fold) in sterilized distilled water. Then, 100 µL of each dilution was placed on YEP plates containing 10 mM sodium selenite and cultured at 30 °C for 2–3 days. Because colonies with red color indicate those with selenite reduction and Se^0^ formation, individual colonies with red color were picked and subcultured on new media to obtain pure cultures. Among monocultures, isolate 801 (named YC801) was chosen for this study, based on its high growth rates and capacity to reduce selenite to red elemental Se.

To identify isolate YC801, biochemical and physiological analyses were conducted using standard methods [49]. Genomic DNA extraction, *16S rRNA* gene fragment amplification, and sequencing of isolate YC801 were performed as described by Huang et al. [60]. Then, the *16S rRNA* gene sequence was compared with those available on the EzBioCloud server [61]. A Maximum Likelihood tree was constructed using MEGA 7.0 software and bootstrap confidence values were obtained with 1000 re-samplings [62]. The resulting sequence was deposited to the GenBank database under the accession number MK071741.

### 3.3. Assessment of Sensitivity towards Selenite

To determine the minimum inhibitory concentration (MIC) of selenite, isolate YC801 was cultured in YEP medium at 30 °C on a shaker (180 rpm) for 24 h. Then, cells were harvested (10 min, 5000× *g*) and resuspended in fresh YEP medium. Finally, aliquots of cell suspensions were added to the flasks (1%, *v*/*v*) containing 10 mL of fresh YEP medium with increasing concentrations of SeO_3_^2−^ (0 to 500 mM). The first concentrations used were 1, 3, 5 and 10 mmol/L. Between 10 and 500 mmol/L, the SeO_3_^2−^ concentration was increased in 20 mM increments. After they had been incubated for 24 h at 30 °C, 100 µL aliquots of culture cells were spotted onto YEP agar plates, and then cultured for an additional 24 h at 30 °C to determine the minimal inhibitory concentration of SeO_3_^2−^ for bacterial isolate YC801.

### 3.4. Quantification of Selenite Reduction and Formation of Se^0^

Reduction efficiency tests of isolate YC801 were performed in 500 mL flasks containing 200 mL of YEP medium spiked either with 1.0 or 5.0 mM selenite. Aliquots from stationary-phase cultures of isolate YC801 were added to each flask to reach a final optical density of 0.01. Bacterial cultures were incubated at 30 °C under aerobic conditions on a rotary shaker incubator (180 rpm).

#### 3.4.1. Dynamics of Microbial Growth after Exposure to SeO_3_^2−^

To elucidate the growth dynamics of isolate YC801, 100 µL of isolate YC801 culture was sampled every 6 h from each flask. Then, all samples were serially diluted with sterile ddH_2_O and spotted onto YEP agar plates. After these cultures were incubated at 30 °C for 24 h, time courses of bacterial growth were obtained by assessing colony-forming units per milliliter (CFU/mL). Isolate YC801 cultured in YEP medium without selenite was used as a negative control.

#### 3.4.2. Assessment of SeO_3_^2−^ Bioreduction Efficiency

To evaluate SeO_3_^2−^ bioreduction efficiency, 10 mL of bacterial culture was sampled every 6 h from each flask and the residual selenite in the medium was determined using inductively coupled plasma optical emission spectrometry (ICP-OES, Thermo Fisher Scientific, Waltham, MA, USA), as described by Nawaz et al. [63] with minor modifications. Briefly, each sample was centrifuged at 12,000× *g* for 20 min. After centrifugation, the supernatant was transferred to a new tube and passed through a 0.22-μm filter for further residual selenite analysis, while the pellet (primarily bacterial cells and elemental selenium) was collected for subsequent Se^0^ content analysis. For residual selenite concentration analysis, 300 µL of filtered supernatant was digested overnight in 3 mL concentrated HNO_3_, then passed through the 0.22-μm filter. Subsequently, samples were diluted to the appropriate selenium concentration and subjected to ICP-OES analysis. The wavelength 203.985 nm was selected for selenium concentration determination.

#### 3.4.3. Se^0^ Content Analysis

The spectrophotometric method was applied to study the Se^0^ content, as described by Khoei et al. [40] with minor modifications. To construct the calibration curve, selenite solutions of 1–10 µmol were reduced by 25 µmol HN_2_OH·HCl, and the intensity of red Se^0^ produced by the reduction was measured at a wavelength of 490 nm. To determine Se^0^ content, the pellets collected from the previous centrifugation step were washed three times with 10 mL of 1 M NaCl to remove selenite contamination, then subjected to sonication as described by Khoei et al. [39]. Once sonicated, the pellets were harvested by centrifugation (12,000× *g* for 10 min), then dissolved in 10 mL of 1 M Na_2_S. Finally, each sample was centrifuged at 12,000× *g* for 20 min to separate the cellular fraction from the supernatant, and the absorption of the supernatant (the reddish solution) was measured via spectrophotometric analysis at 490 nm.

### 3.5. Localization of SeNPs

To determine the location of SeNPs within the bacterial cells, isolate YC801 was grown either in the YEP medium only (control) or in the YEP medium supplemented with 5.0 mM Na_2_SeO_3_ at 30 °C. Then, the bacterial cultures were observed by SEM and TEM.

#### 3.5.1. TEM Analysis

For TEM observation, 24-h cultures of bacteria grown in YEP medium, with and without Na_2_SeO_3_, were harvested by gentle centrifugation (5000× *g* for 5 min). Then, the pellets were mixed with a pre-cooled fixative solution (2% glutaraldehyde in 0.1 M phosphate-buffered saline (PBS), pH 7.4) and fixed at 4 °C for 10 min; this was followed by centrifugation at 5000× *g* for 3 min to avoid mechanical extrusion. Subsequently, the supernatant was fixed overnight at 4 °C and examined at 80.0 KV with at a Hitachi HT-7700 (Tokyo, Japan) transmission electron microscope.

#### 3.5.2. SEM Analysis

For SEM observation, 24-h cultures of bacteria grown in YEP medium, with and without Na_2_SeO_3_, were harvested by gentle centrifugation (5000× *g* for 5 min). Then the pellets were washed three times with 0.1 M PBS (pH 7.4), and fixed with 2.5% glutaraldehyde overnight at 4 °C. Subsequently, the pellets were rinsed in 0.1 M PBS (pH 7.4) three times for 20 min each, and then dehydrated by using an ethanol gradient (30, 50, 70, 80, 95, 100%) for 15 min per gradient step. Finally, each sample was dried by the critical-point drying method, sputter-coated with gold, and examined with a Hitachi S4800 scanning electron microscope (Tokyo, Japan).

### 3.6. Preparation and Characterization of SeNPs

#### 3.6.1. Preparation of Biogenic SeNPs

In order to obtain SeNPs, the isolate YC801 was grown in YEP medium supplemented with 5.0 mM selenite, in a rotary shaker (180 rpm) at 30 °C overnight. After incubation, the bacterial cultures were centrifuged at 12,000× *g* for 10 min and the pellets were collected. The pellets were rinsed twice with 0.9% NaCl and resuspended in 20 mL of Tris-Cl buffer (50 mM, pH 8.2). Then, the samples were subjected to ultra-sonication treatment, and SeNPs were harvested as described by Wang et al. [41]. 

#### 3.6.2. DLS Analysis

A Zen 3600 Zetasizer Nano-ZS (Malvern Instruments Ltd., Worcestershire, UK), equipped with a 633-nm helium-neon laser light source (4.0 mW), was used for DLS analyses of the biogenic SeNPs. Suspensions of SeNPs were diluted 1:10–1:20 to achieve concentrations suitable for the instrument before beginning measurement. The mean size distribution of SeNPs was determined using the software provided by Malvern Instruments.

#### 3.6.3. SEM and EDX Analyses

To identify the morphology and chemical elements that constitute the SeNPs, they were analyzed by SEM and EDX. Before analysis, SeNP suspensions were dried by a vacuum freeze-drying system (SJIA-10N, Ningbo YinZhou Sjia Lab Equipment Co., Ltd., YinZhou, China). Then, the dried selenium particles were examined by a scanning electron microscope with energy-dispersive X-ray spectra (Hitachi S4800, Tokyo, Japan).

#### 3.6.4. FTIR Measurement

SeNPs were characterized by FTIR analysis. First, a drop of the SeNP suspension was settled onto a BaF_2_ support and dried by a vacuum freeze-drying system (SJIA-10N, Ningbo YinZhou Sjia Lab Equipment Co., Ltd., YinZhou, China). Then, mid-infrared spectra (4000–400 cm^−1^) were taken using a Nicolet IS10 spectrometer (Thermo Fisher Scientific, Waltham, MA, USA) employing the transmission mode. 

### 3.7. Detection of the Localization of Selenite Reduction Activity

In order to determine where selenite reduction occurs within the cell compartments and clarify the reduction mechanism, selenite reduction was assayed within different fractions including the cytoplasm, periplasm, membrane, and EPS fractions, as well as in the culture supernatant.

#### 3.7.1. Protein Extraction

To obtain different protein fractions, isolate YC801 was grown to exponential phase (20 h of incubation) and centrifuged at 12,000× *g* at 4 °C for 10 min to obtain bacterial cell pellets. Then, the pellet was washed twice with 0.9% NaCl and pelleted again before protein extraction. Finally, the respective periplasmic, cytoplasmic, and membrane fractions were extracted, using the process described by Lampis et al. [40]. In addition, the total protein content of the three protein fractions was determined by the Bradford method, with bovine serum albumin as the standard.

#### 3.7.2. EPS Extraction

The method described by Lampis et al. [18] was adopted for the extraction of EPS. Briefly, after 5 days of incubation, cultured bacteria were centrifuged at 12,000× *g* for 30 min at 4 °C to separate cells from the culture supernatant. Then, the supernatant was filtered through a 0.22 µm filter and mixed with an equal volume of pre-cooled ethanol. After precipitation at −20 °C overnight, the precipitated EPS fraction was harvested by centrifugation at 12,000× *g* for 30 min at 4 °C, then dissolved in sterile ddH_2_O.

#### 3.7.3. Supernatant Preparation

For supernatant preparation, isolate YC801 was cultured at 30 °C for 24 h. After incubation, the bacterial cultures were centrifuged at 12,000× *g* for 10 min to separate cells from the culture supernatant. Then, the supernatant was filtered through a 0.22 µm filter for later use. 

#### 3.7.4. Selenite Reduction Assay on Different Subcellular Fractions 

Selenite reduction activities of individual fractions of bacterial cultures were measured as described by Khoei et al. [39] with minor modifications. A 96-well plate was used for testing reduction activity. Briefly, each well was filled with the following mixture: 100 µL of the separated protein (adjusted to 2 mg/mL), EPS, or supernatant samples; 88 µL of McIlvaine buffer; 10 µL of sodium selenite solution (a final concentration of 5.0 mM); and 2 µL of NADH (electron donor, final concentration 2.0 mM). Then, the mixture was incubated at 30 °C for at least 72 h. The experiment was also conducted with NADPH as an alternative electron donor. Three negative controls were prepared at the same time: a well without selenite, a well without an electron donor, and a well without an intracellular fraction (or EPS or supernatant). 

### 3.8. qPCR

To identify the candidate enzymes that might be responsible for SeO_3_^2−^ reduction and SeNPs production, the mRNA expression of several candidate enzymes, including glutathione synthase (*gsh*B) and nitrite reductase (NAD(P)H) (*B9475_02395*) under selenite treatment were detected using qPCR. The gene-specific primers are listed in Table 2 with the housekeeping gene (16S rRNA) used as a reference.

For RNA extraction, isolate YC801 was cultured in YEP medium (control) or YEP medium supplied with 5.0 mM Na_2_SeO_3_ for 20 h at 30 °C. After incubation, bacterial cultures were harvested by centrifugation (5000× *g* for 5 min) and total bacterial RNA was extracted using an E.Z.N.A. bacterial RNA kit (Omega Bio-Tek, Norcross, GA, USA), in accordance with the manufacturer’s protocol. cDNA was then synthesized using TransScript One-Step gDNA Removal and cDNA Synthesis SuperMix (TransGen Biotech, Peking, China) and subjected to qPCR analysis using a QuantiFast SYBR Green PCR kit (Qiagen, Hilden, Germany), as described by Wang et al. [41]. The RNA transcription levels were calculated in terms of the change with respect to untreated cells (control), using the 2^−ΔΔ*C*t^ method. Each sample was analyzed in triplicate, and three independent tests were conducted (a total of nine analyses of each sample). The gene-specific primers are listed in Table 2, with the housekeeping gene (*16S rRNA*) used as a reference.

### 3.9. Statistical Analysis

All analyses were performed in triplicate, and the data presented are the mean values of three independent sets. Each value was expressed as the mean ± standard deviation (SD). One-way analysis of variance (ANOVA) at a 95% confidence level followed by Tukey’s test was used to evaluate whether the means were significantly different from each other. The level of significance was set at *p* < 0.05.

## 4. Conclusions

In conclusion, the gut bacterium, *P*. *mirabilis* YC801, was isolated from the gut of the adult form of *M. alternatus* and exhibited high resistance towards SeO_3_^2−^ (MIC^Se^ of 100 mM). To the best of our knowledge, this is the first study to demonstrate that *P. mirabilis* is able to perfectly transform toxic SeO_3_^2−^ to Se^0^ with a high reaction rate in a liquid growth medium under aerobic conditions. In doing so, this strain produces SeNPs of spherical shape with average dimensions of 178.3 ± 11.5 nm and the SeNPs localized mainly in the extracellular space. In vitro biocatalytic selenite reduction activity assays revealed that SeNPs formation can be tentatively attributed to cytoplasmic enzymatic activation mediated by electron donors (NADPH or NADH). Considering the high potential of biogenic SeNPs in the fields of nanobiotechnology, medicine, and environmental biotechnology, *P. mirabilis* YC801 represents an attractive biological resource of selenium nanoparticles. However, additional studies are needed to further clarify the specific mechanisms of SeNPs synthesis and release, as well as the properties of these biogenic SeNPs.

## Figures and Tables

**Table 1:** Biochemical and physiological characteristics of the bacterial isolate YC801.

Characteristic	Result	Characteristic	Result
Gram-staining	−	Enzyme activities:	
Nitrite reduction	+	α-Glucosidase	+
Motility	+	Protease	−
Oxidase	−	Utilization of:	
Catalase	+	Maltose	−
Indole test	+	Lactose	−
Nitrate reduction	+	Methyl β-D-glucoside	−
Urease	+	*cis*-Aconitic acid	+
Hydrolysis of:		Raffinose	−
Starch	−	L-Arabinose	+
Gelatin	+	L-Histidine	+
Hydrogen sulfide test	+	Trehalose	+

Positive result or weakly positive (+), Negative result (−).

**Table 2:** Primers for targeting genes.

Target Gene	Primer Sequence	Product Size (bp)
Glutathione synthetase (A0A1Z1SXV1)	Forward 5′-CACGCCAAGTAGCACCT-3′ Reverse 5′-TCAATCTCACGAGCACAA-3′	118
Thioredoxin reductase (B4EV88)	Forward 5′-AACCGACCTTTCCGCCTAT-3′ Reverse 5′-CCACCACCGACAACAGCA-3′	191
Nitrite reductase (NAD(P)H) (A0A205JSA1)	Forward 5′-GCAAATCGCTCAAGAATA-3′ Reverse 5′-CACCAACTACTGCCTACA-3′	186
Glutathione reductase (B4EZ75)	Forward 5′-TAAATGCGTTAGGGAGTG-3′ Reverse 5′-CTGTAGCAGGTTCACGAC-3′	246
Fumarate reductase subunit D (B4EWY4)	Forward 5′-CAGGTGGTATGTGGAGTG-3′ Reverse 5′-AAATCGTGCAACGTATGG-3′	208
Fumarate reductase subunit C (B4EWY5)	Forward 5′-AACTGGTGGACGAAACTC-3′ Reverse 5′-GCGATAAGGGTCACAATA-3′	194
Thioredoxin (B4F1V4)	Forward 5′-CGTGCTCGTTGATTTCT-3′ Reverse 5′-GGTGCTGTCGCAGGGTT-3′	138
Sulfite reductase [NADPH] flavoprotein alpha-component (B4F235)	Forward 5′-ATTATCCCGCCACGAAGA-3′ Reverse 5′-AAGCGATGGAGTAAAGACG -3′	173
16S r RNA	Forward 5′-AGAGTTTGATCCTGGCTCAG-3′ Reverse 5′-CTGCTGCCTCCCGTAGGAGT-3′	330

## Abbreviations

SeNPs	Se nanoparticles
Se^0^	Elemental Selenium
TEM	Transmission electron microscopy
SEM	Scanning electron microscopy
EDX	Energy dispersive X-ray analysis
MIC	Minimum inhibitory concentration
NADH	Nicotinamide adenine dinucleotide
NADPH	Nicotinamide adenine dinucleotide phosphate
qPCR	Real-time quantitative PCR
