# Correction: Wang, Y.T., et al. Selenium Nanoparticle Synthesized by *Proteus mirabilis* YC801: An Efficacious Pathway for Selenite Biotransformation and Detoxification. *Int. J. Mol. Sci.* 2018, *19*, 3809

**DOI:** 10.3390/ijms21072638

**Published:** 2020-04-10

**Authors:** Yuting Wang, Xian Shu, Jinyan Hou, Weili Lu, Weiwei Zhao, Shengwei Huang, Lifang Wu

**Affiliations:** 1 Key Laboratory of High Magnetic Field and Ion Beam Physical Biology, Hefei Institutes of Physical Science, Chinese Academy of Sciences, Hefei 230031, Anhui, China; wangyuting2006@outlook.com (Y.W.); sx360775419@gmail.com (X.S.); jyhou@ipp.ac.cn (J.H.); luweili@ahmu.edu.cn (W.L.); annyzhao@ipp.ac.cn (W.Z.); 2 The Sericultural Research Institute, Anhui Academy of Agricultural Science, Hefei 230061, Anhui, China; 3 School of Life Sciences, University of Science and Technology of China, Hefei 230026, Anhui, China; 4 Key Laboratory of Environmental Toxicology and Pollution Control Technology of Anhui Province, Hefei Institutes of Physical Science, Chinese Academy of Sciences, Hefei 230031, Anhui, China; 5 Anhui Key Laboratory of Bioactivity of Natural Products, School of Pharmacy, Anhui Medical University, Hefei 230032, Anhui, China

The authors wish to make the following corrections to this paper [1]:

**Abstract:** The phrase concerning FTIR spectroscopic analyses in the abstract should read as follows: Finally, the Fourier transform infrared spectral analysis confirmed the presence of proteins and carbohydrates on the surface of SeNPs.

## 1. Change in Main Body Paragraphs

The authors are sorry to report that some of the FTIR data reported in their recently published paper [1] were incorrect. We have recently been made aware by Prof. Alexander A. Kamnev (Institute of Biochemistry and Physiology of Plants and Microorganisms, Russian Academy of Sciences, Saratov) that the FTIR spectrum was presented and interpreted erroneously because it was illustrated with the vertical (intensity) axis “Transmittance“ (with «peak maxima» directed downwards), while the spectrum itself was plotted as if with the vertical (intensity) axis “Absorbance” (with the real absorption peaks directed upwards). Consequently, the authors wish to make, at this time, the following corrections to the paper:

2.4.2. Fourier Transform Infrared (FTIR) Spectroscopic Analysis

The Fourier transform infrared (FTIR) spectrum of the SeNPs produced by YC801 is presented in Figure 7, and the various absorption bands in the FTIR spectrum and the corresponding assignments are given below. The band recorded at 3290 cm^−1^ can be assigned to the NH stretching vibrations with intermolecular bonds over the broad O-H stretching envelope of the alcohol associated groups (and possible bound water traces), while the peak at 3070 cm^−1^ is assigned to the amide A band, typical of proteins. Different typical C-H stretching vibrations in the aliphatic groups appeared at 2960–2850 cm^−1^. The peak at 1660 cm^−1^ indicates the presence of the amide I band, accompanied with the weaker peak at 1530 cm^−1^ of amide II in proteins. The spectra also showed a CH_2_ scissoring band of the aliphatic groups and carbohydrates at 1456 cm^−1^. Furthermore, peaks at 1400, 1236, and 1075 cm^−1^ could mainly be attributed to the existence of the C-H bending, amide III (proteins)/CH_2_ wagging vibrations, and carbohydrate C-O stretching vibrations, respectively. Finally, the presence of a peak at 794 cm^−1^ can be attributed to the O-H translational vibrations. 

Thus, the present results clearly indicate the presence of organic residues, such as carbohydrates and proteins, on the surface of the SeNPs produced by YC801. Lampis et al. [40] found that various organic residues were present on the surface of biogenic SeNPs produced by *S. maltophilia* but completely absent in SeNPs synthesized chemically. It suggested these biochemicals may be involved in the processes of formation and/or stabilization of biogenic SeNPs. Debieux et al. [47] also reported that Se nanospheres produced by *Thauera selenatis* were stabilized under the presence of a protein of approximately 95 kDa – Se factor A (SefA), while Dobias et al. [48] identified four proteins (AdhP, Idh, OmpC, and AceA) that bound specifically to bio-SeNPs, which played a critical role in controlling the particle size and morphology of the SeNPs. Therefore, our findings not only indicate that bacterial proteins may be involved in the selenite reduction but also corroborate their involvement in the processes of synthesis and stabilization of SeNPs.

## 2. Change in Figure

The author wishes to make the following correction to this paper [1]. Due to mislabeling, replace:

with the corrected Figure 7 (Figure 1):

These changes have no material impact on the conclusions of our paper. We apologize for any inconvenience caused to the readers by these changes.

## Figures and Tables

**Figure 7:**
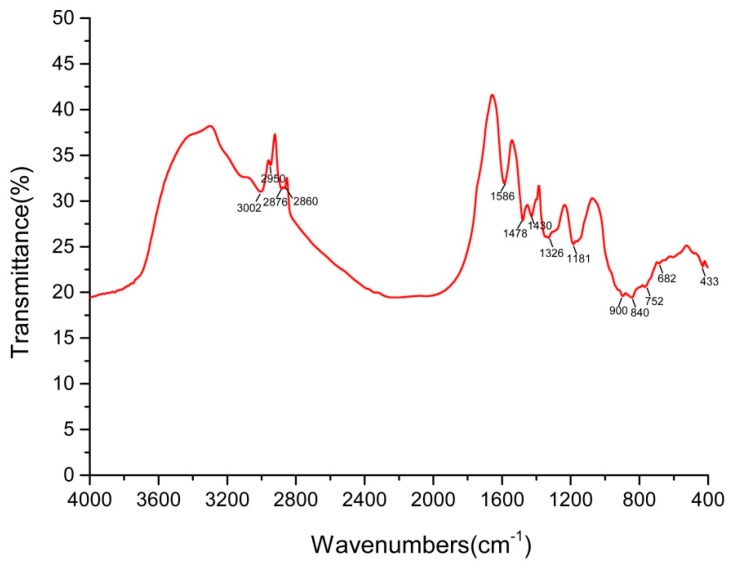
The FTIR spectrum of SeNPs synthesized by isolate YC801.

**Figure 1:**
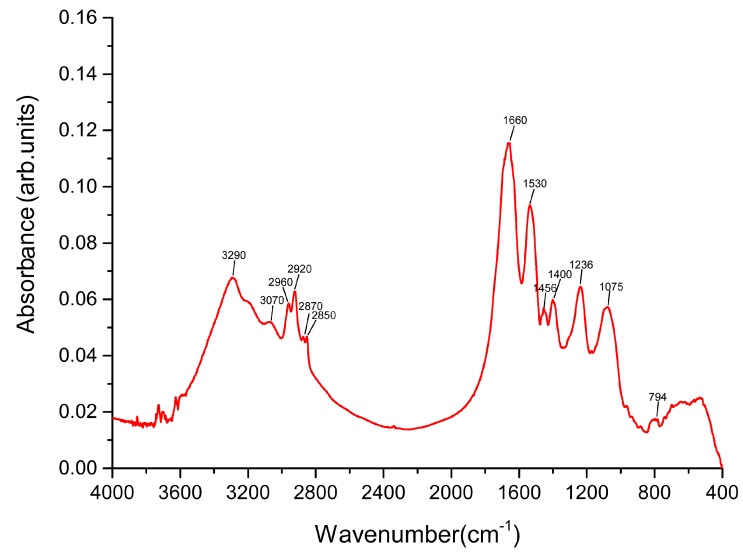
The FTIR spectrum of SeNPs synthesized by isolate YC801.
